# The Impact of Experimental Preconditioning Using Vascular Endothelial Growth Factor in Stroke and Subarachnoid Hemorrhage

**DOI:** 10.1155/2013/948783

**Published:** 2013-03-24

**Authors:** Sven Oliver Eicker, Moritz Hoppe, Nima Etminan, Stephan Macht, Jason Perrin, Hans-Jakob Steiger, Daniel Hänggi

**Affiliations:** ^1^Department of Neurosurgery, Heinrich-Heine-University, Medical Faculty, 40225 Düsseldorf, Germany; ^2^Department of Neurosurgery, University Medical Center, Hamburg-Eppendorf, 20246 Hamburg, Germany; ^3^Department of Radiology, Heinrich-Heine-University, Medical Faculty, 40225 Düsseldorf, Germany

## Abstract

Vascular endothelial growth factor (VEGF) stimulating angiogenesis was shown to be a potential novel therapeutic approach for the treatment of ischemic vascular diseases. The goal of the present study was to examine whether transfection of VEGF before occurrence of major stroke (part I) and cerebral vasospasm after experimental subarachnoid hemorrhage (SAH; part II) develops neuroprotective qualities. A total of 25 (part I) and 26 (part II) brains were analyzed, respectively. In part one, a significant reduction of infarct volume in the VEGF-treated stroke animals (43% reduction, *P* < 0.05) could be detected. In part two, significant vasospasm was induced in all hemorrhage groups (*P* < 0.02). Analyzing microperfusion, a significant higher amount of perfused vessels could be detected (*P* < 0.01), whereas no significant effect could be detected towards macroperfusion. Histologically, no infarctions were observed in the VEGF-treated SAH group and the sham-operated group. Minor infarction in terms of vasospasm-induced small lesions could be detected in the control vector transduced group (*P* = 0.05) and saline-treated group (*P* = 0.09). The present study demonstrates the preconditioning impact of systemic intramuscular VEGF injection in animals after major stroke and induced severe vasospasm after SAH.

## 1. Introduction

Cerebral vasospasm and delayed cerebral ischemia contribute the major part of secondary morbidity and mortality after severe subarachnoid hemorrhage (SAH) [1–5]. Despite the current treatment strategies, the rate of related permanent disability is estimated at 10% to 20% [6–9].

Vascular endothelial growth factor (VEGF) is involved in neurogenesis, inhibition of apoptosis, learning, and memory [10]. It can directly promote neuroprotection, but first of all VEGF is the main factor responsible for angiogenesis whereby an indirect neuroprotection is discussed. VEGF expression is increased during cerebral ischemia in humans and animals [11]. However, endogenous VEGF seems to be insufficient to protect the brain from ischemic injury completely. Interestingly, it could be shown that exogenous administrated VEGF induces angiogenic changes that result in a reduction of cerebral ischemic injury [12, 13]. For this reason VEGF was adopted as a potential novel therapeutic approach for the treatment of ischemic vascular disease, particularly in ischemic stroke [14–18].

The aim of the present experimental study was to examine the effect of systemic overexpression of VEGF prior to stroke and SAH with regard to cerebral infarction, vasospasm, and perfusion.

## 2. Material and Methods

This study was carried out in strict accordance with the recommendations in the Guide for the Care and Use of Laboratory Animals of the National Institutes of Health. The experimental study was reviewed and approved by the local Committee for Animal Experimentation, Recklinghausen, Germany (approval no. 8.87-50.10.34.08.246). All invasive procedures were performed under general anesthesia with intraperitoneal application of xylazine hydrochloride and ketamine, and all efforts were made to minimize suffering. The animals were housed under a light/dark cycle with free access to food and water.

### 2.1. Construction of Vectors and Transduction

DNA transduction was performed with a VEGF-containing expression vector. The initial vector (pcDNA3.1/His B/; Invitrogen, Karlsruhe, Germany) was first digested with EcoRI. A VEGF clone in EcoRI (descending from a pLEN/VEGF vector, kindly provided from Max-Planck-Institute, Planegg-Martinsried, Germany) was inserted. Control animals were injected with an empty expression vector (pcDNA3.1/His B; Invitrogen, Karlsruhe, Germany). Vector integrity was confirmed by sequence analysis. Large-scale preparation of plasmid DNA was performed with the EndoFree GigaPrep (Qiagen, Hilden, Germany). DNA was solved in 0.9% sterile saline and stored in aliquots at −20°C. 

Weekly gene transfers into both anterior tibial muscles were performed thrice with 100 *μ*g DNA per leg in a volume of 50 *μ*L normal saline. 

### 2.2. Transient Middle Cerebral Artery Occlusion

Part one determined the effect of VEGF in stroke protection in order to verify the effect of VEGF in major stroke. Three groups of male Wistar rats weighting 250 to 275 g (*n* = 32) received intramuscular injections of VEGF vector, control vector, or saline for three times at intervals of seven days. Seven days after the last gene transfer, the rats underwent 45 minutes of transient middle cerebral artery occlusion (tMCAO) under continuous monitoring of laser Doppler flow (Moor Instruments, Axminster, UK) [19]. Body temperature was maintained at 37.0 ± 0.5°C. Reperfusion was performed by removing the filament. Animals were sacrificed after 24 h reperfusion time.

### 2.3. Rat Double SAH Model

In part two a total of 80 male Wistar rats weighting 250 to 275 g were used. The animals were randomized to four groups: group (1) receiving intramuscular injections with a plasmid-containing VEGF, group (2) receiving a VEGF free plasmid, group (3) the saline group, and group (4) the sham-operated group.

In groups 1 and 2 the VEGF or VEGF free vector was injected 21, 14, and 7 days before the induction of vasospasm. 

One week after the last gene transfer (VEGF vector, control vector, and sodium) vasospasm was induced by double blood injection into the cisterna magna as described previously [20–22]. Animals were positioned prone, and the atlantooccipital membrane was surgically exposed. The cisterna magna was punctured under microscopic view using a 27-gauge cannula, and 0.2 mL of cerebrospinal fluid was aspirated first and followed by injection of 0.2 mL of autologous blood. The animals were then placed head down for 10 minutes to avoid leakage of injected blood, and the operation wound was closed. The procedure was repeated on day 2. In animals belonging to the sham-operated group, the atlantooccipital membrane was exposed; 0.2 mL of cerebrospinal fluid was aspirated and reinjected. The animals were positioned head down for 10 minutes, and the operation wound was closed. This procedure was repeated on day 2. During the procedure the body temperature was controlled and maintained at 37.0 ± 0.5°C.

The neurological condition was assessed daily according to a modified Bederson grading scale circling to one side [23]. 

### 2.4. Cerebral Angiography and Evaluation

Angiography was performed on day five after double direct blood injection into the cisterna magna. 

The angiographic studies were performed under intraperitoneal general anesthesia as described above. After positioning the animals supine, a cervical midline incision was made to expose the common carotid artery bilaterally. As described previously the artery was tapped using a small cannula attached to a microcatheter (27-gauge needle, Prowler 14 microcatheter; Cordis Endovascular, Miami Lakes, FL, USA), and the angiography was performed under automated, controlled injection of a total of 0.1 mL of contrast agent (Ultravist 300; Schering AG, Berlin, Germany; Integris Allura; Philips Medical Systems, Best, The Netherlands) [20]. The angiography was repeated up to four times to achieve best quality. On the one hand digital imaging was measured, software based, to evaluate the reduction of large vessel diameter as described before [24]. On the other hand a visualization of low-density structures in given regions of interest (RIO) was determined in order to obtain information concerning perfusion by the use of minimum intensity projection (MinIP) [25]. A MinIP in time direction from the beginning arterial phase to the parenchyma phase was carried out. The algorithm uses all the data by projecting the volume of interest into a viewing plane. Before contrast agent arrived, an individual baseline image was determined and filtered to reduce background information (OsiriX v. 3.8.1, http://www.osirix-viewer.com/). Major ROIs (including angiographically visible major vessels, e.g., A. carotis interna) and smaller cortical ROIs (without angiographically visible major vessels, e.g., area between A. carotis interna beneath the junction of A. cerebri media) were defined. The resulting bidimensional image represents the contrast-perfused vessels/tissue. Measurement of average grey levels in the above-defined ROIs represents a higher or lower perfusion. These values of minimal intense projection are inversely correlated with the amount of perfused vessels.

The animals were sacrificed after the angiography by intraperitoneal injection of a lethal dose of sodium pentobarbital (200 mg/kg body weight, Sanofi-Aventis, Frankfurt, Germany).

### 2.5. Histology

A 12 *μ*m microtome was used for cresyl violet staining and hematoxylin and eosin as well as for TUNEL analysis. Coronal sections of the frontal, parietal, and occipital brain were taken to detect morphological alterations in terms of ischemic lesions. In part one recording of all sections was obtained by a digital camera. Infarct area and total area of the brain were outlined manually and volume calculated, software based, in mm^3^ (Leica QWin, Leica, Germany). In part two the infarctions in each section were assessed and divided into three groups as previously published: (1) no infarction, (2) minor infarction, and (3) territorial infarction [26]. For detection of apoptotic cells in part one, an in situ cell death detection kit (Roche Diagnostics, Mannheim, Germany) based on terminal deoxynucleotidyl transferase-mediated deoxyuridine triphosphate nick end-labeling (TUNEL) technique was used. 

## 3. Results

### 3.1. Part One: Attenuated Ischemic Brain Injury after Transient Focal Cerebral Ischemia

**Figure 1:**
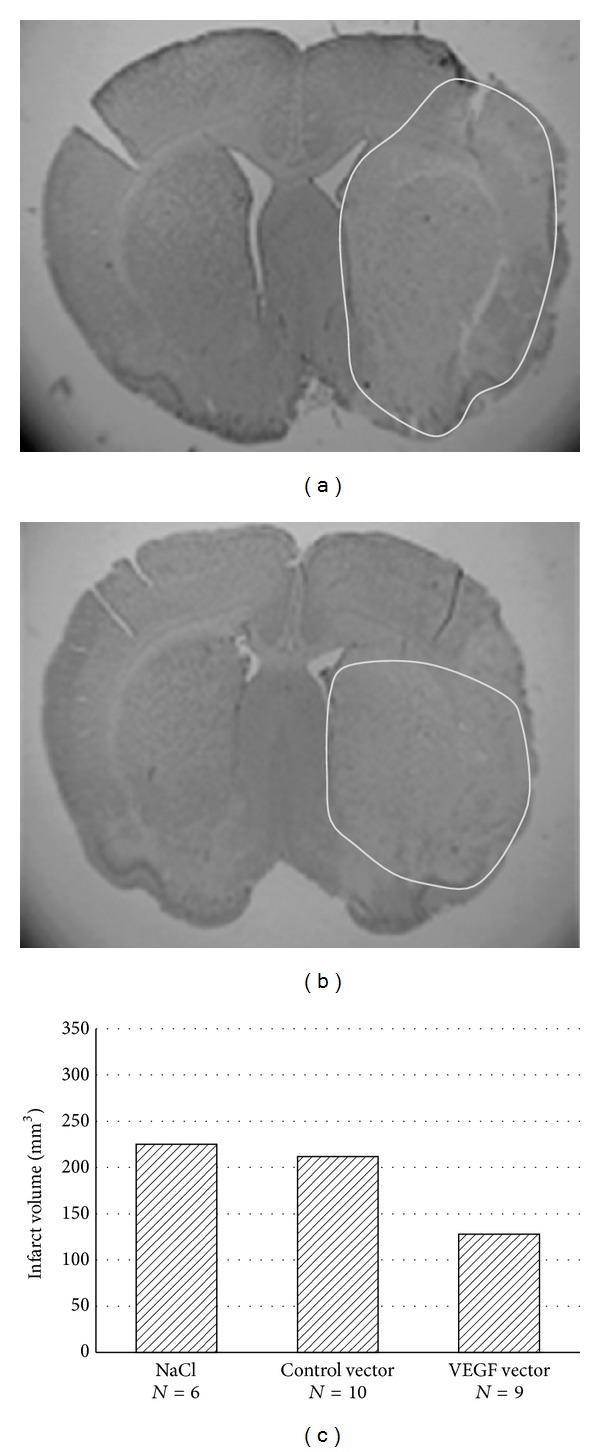
Cresyl violet staining after 45 minutes of tMCAO and 24 h reperfusion time. (a) Distinct ischemic brain injury after i.m. injection with saline. (b) Attenuated ischemic brain injury after i.m. injection with VEGF. (c) Infarct volume in the three different groups in mm^3^.

In part one VEGF in major stroke showed a significant reduction of infarct volume in the VEGF-treated animals (43% reduction, *P* < 0.05, Mann-Whitney *U* test, Figure 1). Five animals passed away due to subarachnoid hemorrhage and two as the result of stroke. 25 brains were analyzed (9 in the gene-transferred group, 10 in the control group, and 6 in the saline group). TUNEL staining showed no significant differences in the three groups.

### 3.2. Part Two

A total of 80 animals were examined. 24 animals died immediately after the double hemorrhage injection and 12 during the course of the experiment. The clinical evaluation revealed no delayed neurological deficit over the 5-day observation period in terms of hemiparesis. All animals were sacrificed on day 5 after the initial bleeding.

#### 3.2.1. Angiographic Analysis of Macroperfusion

Overall, 176 angiographic examinations were performed in 44 animals belonging to the 4 experimental groups. Angiographic evaluation was not possible in 18 animals as a result of technical problems or mortality from the angiography itself. 96 series in 26 animals were technically sufficient. Of these, the angiogram with the highest contrast of each animal was chosen for further evaluation by an independent observer (SM) blinded to the groups.

**Figure 2:**
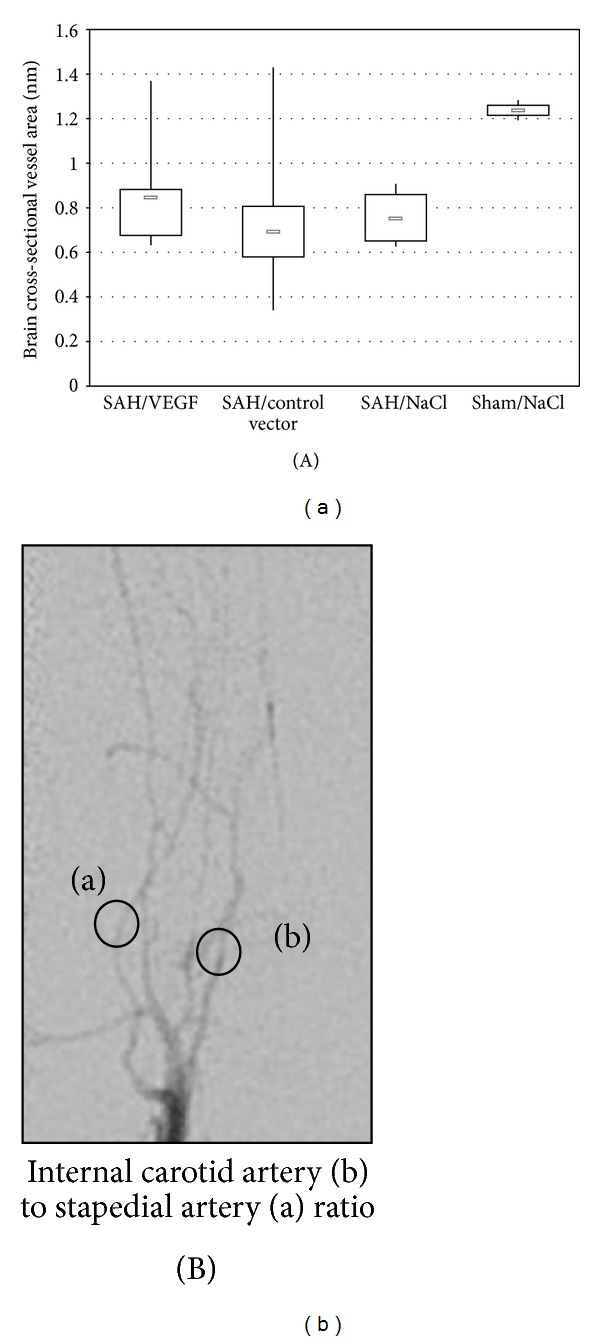
(A) The brain cross-sectional vessel area in mm in the three SAH groups and the sham group. Displayed are the lowest, highest, and median values of brain cross-sectional vessel area (in mm), with upper (75%) and lower (25%) quartiles. (B) Digital subtraction angiography of rat number 11 demonstrating the internal carotid artery (b) to stapedial artery (a) ratio.

Statistically significant reduction of arterial diameter was induced with the double hemorrhage model (SAH groups compared with sham group, *P* < 0.02, Figure 2). Among the three SAH groups, there were no statistically significant differences of relative intracranial filling intensity defined as macroperfusion (SAH/VEGF compared with SAH/control, *P* = 0.56; SAH/VEGF compared with SAH/NaCl, *P* = 0.51, *t*-test).

#### 3.2.2. Angiographic Analysis of Microperfusion

**Figure 3:**
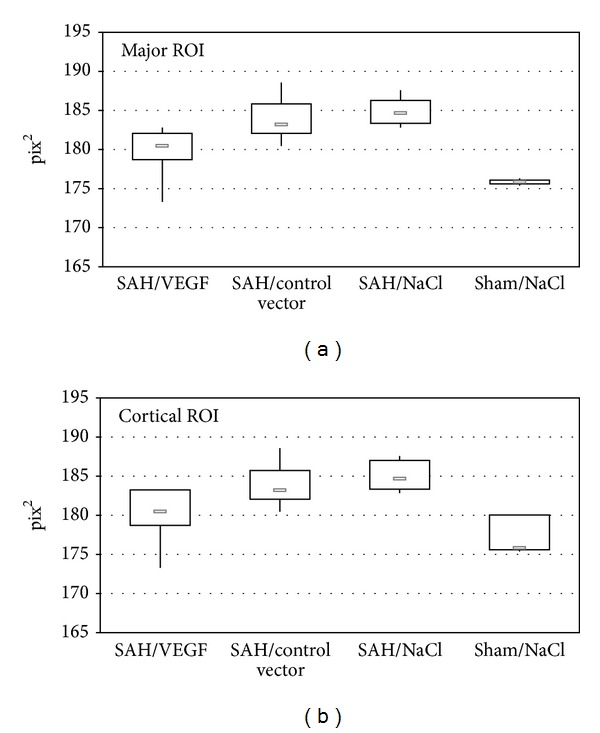
Minimum intense projection of cortical (without angiographic visible vessels) and major ROIs (with angiographic visible vessels): values of minimal intense projection are inversely correlated with the amount of perfused vessels. Displayed are the lowest, highest, and median pix^2^ values, with upper (75%) and lower (25%) quartiles.

In 26 animals, a complete arterial to venous phase (circulation time) was carried out (Figure 3). Comparing the VEGF-treated SAH group (group 1) with the control vector treated SAH group (group 2) and the NaCl-injected SAH group a significant difference was detected (major ROI: *P* < 0.01 and *P* < 0.01; cortical ROI: *P* < 0.02 and *P* < 0.01). Differences between the VEGF-treated and NaCl groups without SAH were not statistically significant (major ROI: *P* = 0.94; cortical ROI: *P* = 0.63). 

#### 3.2.3. Morphological Examination

**Figure 4:**
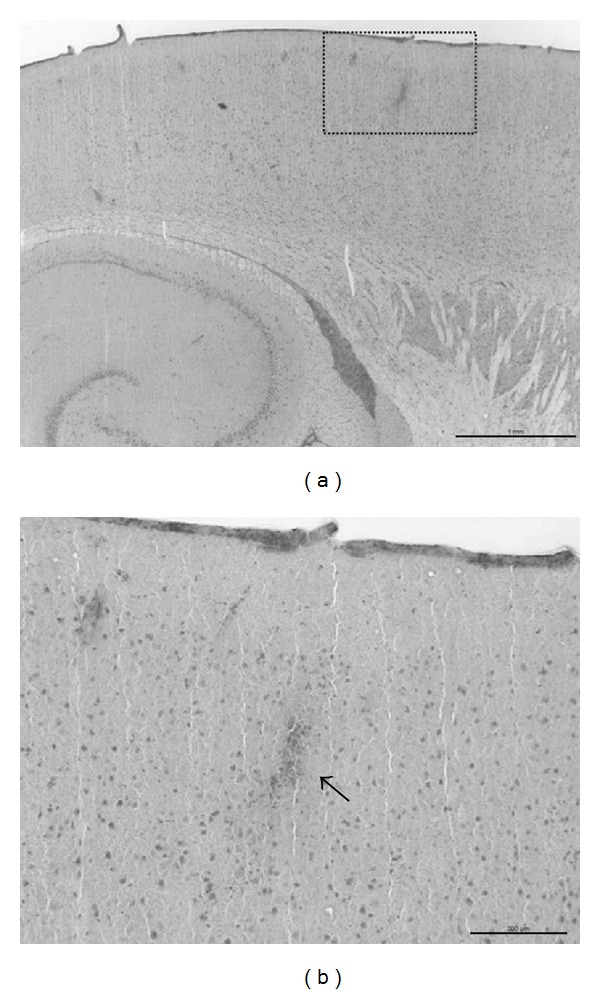
H&E stained section with cortical microinfarct in the cortical layers in a nontreated animal (a). At higher magnification mononuclear infiltration can be distinguished (b, arrow). Scale bar corresponds to 1000 *μ*m (a) and 200 *μ*m (b), respectively.

Macroscopic pathological evaluation revealed clear residuals of SAH in the basal cisterns in 26 animals belonging to the SAH groups and in none of the sham-operated group. Histologically, no infarctions were observed in the VEGF-treated group and the sham-operated group. Minor infarction in terms of vasospasm-induced small lesions could be detected in the control vector transduced group (*P* = 0.05) and saline-treated group (*P* = 0.09) (Figure 4). 

## 4. Discussion

### 4.1. Part One

Analyzing therapeutic effect in experimental cerebral ischemia usually a tMCAO model is used [27, 28]. Therefore the developed VEGF DNA was initially tested in this experiment under well-defined conditions with clearly expected ischemic lesions. Different VEGF levels and accordingly immunohistochemical analysis of VEGF were not carried out, but a distinct effect on the reduction of infarct volume in the VEGF-treated animals could be shown (43% reduction). Based on these results it is assumed that intramuscular injection of VEGF has a neuroprotective effect in cerebral ischemia animal model.

### 4.2. Part Two

#### 4.2.1. SAH Model

The double hemorrhage model was used because of the higher vasospastic impact in comparison to other SAH models as described [29]. As previously published, the highest level of measurable vasoconstriction was induced on day 5 after induction of SAH [20, 30]. Thus, day 5 was chosen for angiographic studies. Mortality rate in the present trial was 30%, which is in line with previously published studies using the double hemorrhage model in rats [20, 31, 32].

#### 4.2.2. Efficacy on Macro- and Microperfusion

Angiographic evaluation of small vessels in rats is complex, and thus different techniques have been reported [33–35]. In the present study, a previously described software-based measurement tool for analysis of small cerebral vessels was used to detect vasospasm [24]. Using this technique, a significant induction of vasospasm in all SAH groups could be detected, which is in line with previously published studies [20].

In the VEGF-treated group, a significant difference of cerebral vessel caliber (cerebral macroperfusion) in relation to the other SAH groups was not measurable. In contrast, the analysis of cerebral microperfusion using the vascular density technique as previously described revealed a significant increase in the VEGF-treated group [25]. One explanation could be the induction of neoangiogenesis due to VEGF. This observation is in line with results after the experimental cerebral ischemia model in animals [35]. Two major limitations have to be mentioned. First, the blood pressure during DSA was not measured and therefore may have affected time to peak and cerebral blood flow values. However, to detect real hyperperfusion or hypoperfusion a perfusion imaging in terms of perfusion CT or perfusion MRI is needed. Second, limitation is the infusion time of contrast agent. Automated injection facilitated a standardized condition in our setting.

#### 4.2.3. Morphological Effects

One issue with experimental models of SAH in small animals is the lack of clear morphological ischemic damage wherefore part one was performed in order to evaluate the effect in major stroke [22]. Similarly, in the present study, only a few ischemic areas in the nontreated SAH groups could be detected. 

### 4.3. Efficiency of Intramuscular VEGF and Limitation of the Trial

Hypoxia itself induces an increase of VEGF expression in ischemic areas of the brain, but this endogenous VEGF secretion is inadequate to entirely protect the brain injury [36]. Based on the significant reduction of infarct volume in part one it is assumed that intramuscular injection of VEGF has a neuroprotective effect in cerebral ischemia animal model.

Short half-life and poor penetration over the blood brain barrier appear to have a lower impact in this model than frequently described [13, 27]. These findings were corroborated by other investigators who verified that high dose of intravenous infusion of VEGF after cerebral embolic ischemia induces leakage of blood brain barrier in the animal model [37]. Controversially, reduction of edema formation after VEGF application in a stroke model despite the leakage of blood brain barrier has been described [38]. The detailed activity of VEGF, besides the known neoangiogenesis and mitogenic activity in stroke and particularly in SAH, remains therefore mostly unknown.

Although VEGF administration appears promising, several disadvantages have to be mentioned. VEGF protein is not stable in vivo, and it has a short half-life. Direct protein implantation into ischemic lesions via sustained release delivery systems or focal virus mediated overexpression might protect the immediately surrounded neuronal tissue, and, therefore, direct implantation seems to be a good approach for defined hypoxic-ischemic brain injuries [27, 39]. In SAH, delayed cerebral ischemia and related infarction are known to occur diffusely. Therefore, recommendation of treatment and the target of therapy should address the whole brain. In consequence, a systemic increase of VEGF using a DNA gene transfer could be discussed as a potential beneficial approach. 

Within the chronic phase of vasospasm, the cerebral circulation adapts to hypoxia with angiogenesis and dilatation of microvessels [15]. Nevertheless, it is suggested that the relation between vasoconstrictive factors (free hemoglobin, activated endothelin-1, and free oxygen radicals) and vasodilatory substances (NO) is disturbed in favor of constrictors, and neoangiogenesis potentially starts too late. In this study no influence of VEGF on the vasoconstrictive or vasodilatory elements could be detected directly. On the opposite, a significant increase of perfusion could be detected. However, a nonsignificant trend to a lesser extend of ischemic lesions in histological examinations could be identified, and a significant increase of microperfusion was detected via an angiographic approach despite proven vasospasm. Against this background the indirect neuroprotection by dint of angiogenesis seems to have a high importance.

The upregulation of VEGF can trigger this angiogenesis in the early period of vasospasm. This means preconditioning the cerebrum to forthcoming ischemic event by improvement of blood supply.

The present study demonstrates the preconditioning impact of systemic VEGF injection on cerebral microperfusion and ischemic lesions in animals after induced severe vasospasm. These results justify further investigations in particular with regard to modus of application, dose of VEGF injection, time interval of preconditioning, immunohistochemical examination (VEGF, CD 34), and detailed measurement of behavior changes.
